# Hemobilia: A Narrative Review of Current Diagnostic Techniques and Emerging Management Strategies

**DOI:** 10.7759/cureus.73009

**Published:** 2024-11-04

**Authors:** Thomas Campos Carmona, Camila Teran Hooper, Vaidarshi Abbagoni, Haya Al Shakkakee, Aarfa Devani, Jonathan D Martinez Illan, Valencia Maryjose, Eduardo E Venegas González, Ilean López Cervantes

**Affiliations:** 1 Medicine, Universidad de Ciencias Médicas, San José, CRI; 2 Medicine, Facultad de Medicina Dr. Aurelio Melean, Universidad Mayor de San Simón, Cochabamba, BOL; 3 Internal Medicine, Hartford Hospital, Bridgeport, USA; 4 Medicine, Al Kindy College of Medicine, University of Baghdad, Baghdad, IRQ; 5 Internal Medicine, Malla Reddy Institute of Medical Sciences, Hyderabad, IND; 6 Medicine, Escuela de Medicina Dr. Jose Sierra Flores, Universidad del Noreste, Tampico Tamaulipas, MEX; 7 Internal Medicine, Universidad Nacional Autónoma de México, Ciudad de México, MEX; 8 Medicine, Universidad Nacional Autónoma de México, Ciudad de México, MEX

**Keywords:** biliary tract hemorrhage, endoscopic management, gastrointestinal tract bleeding, hemobilia, hepatic vascular injury, hepato-biliary, hepato-pancreato-biliary surgery, iatrogenic lesion, quincke's triad, transcatheter arterial embolization

## Abstract

Hemobilia is a relatively uncommon but important cause of gastrointestinal bleeding. It occurs due to abnormal communications between the biliary system and surrounding vasculature, often caused by surgical interventions, trauma, infections, or malignancies. The rise of advanced hepato-pancreato-biliary techniques, including radiofrequency ablation and transjugular intrahepatic portosystemic shunt (TIPS) placement, necessitates careful evaluation for the potential presence of hemobilia during the post-procedural period of these patients. Hemobilia can be difficult to diagnose, as common symptoms like jaundice, abdominal pain, and gastrointestinal bleeding are not always present together. Imaging techniques such as Doppler ultrasound, contrast-enhanced computed tomography (CT), and angiography are critical for identifying the source of bleeding. Treatment typically focuses on achieving hemostasis and ensuring proper bile flow, with options including endoscopic techniques, angiography with transcatheter arterial embolization, and, in severe cases, surgical intervention. This review highlights recent advances in diagnostic and therapeutic approaches, emphasizing the need for early recognition and tailored interventions to improve patient outcomes.

## Introduction and background

Hemobilia, in its simplest form, is defined as the presence of blood within the biliary tract due to an abnormal communication between the bile ducts and the surrounding vasculature [1-4]. Hemobilia, though rare, presents significant diagnostic and therapeutic challenges due to its varied etiology, ranging from trauma to malignancy, it was first documented as early as the 1600s, but its incidence has increased as the number of minimally invasive hepato-pancreato-biliary procedures has risen in recent decades [5]. Francis Glisson, a physician, documented the first known case of hemobilia in 1654 [6]. He described the case of a nobleman who suffered a fatal injury to his right upper quadrant during a sword fight, resulting in massive upper gastrointestinal bleeding and death. During the autopsy, it was discovered that the liver had been lacerated, thus causing massive bleeding which ultimately led to the landmark description of hemobilia [1]. In 1885, Quincke identified the clinical triad of right upper quadrant pain, jaundice, and upper gastrointestinal bleeding, now known as "Quincke's triad" [7,8].

The clinical presentation is determined by its underlying cause. Understanding the causes of hemobilia is crucial for early diagnosis and effective management of this condition, which can have severe consequences if left untreated. While several studies have investigated the various etiologies of hemobilia, such as infections, malignancies, trauma, and iatrogenic causes, there is limited research on the iatrogenic causes, which have become more prevalent in recent years due to widespread interventional procedures, including ultrasound-guided radiofrequency ablation, intrahepatic portosystemic shunt (TIPS) placement, and endoscopic procedures involving the hepatobiliary structures [9]. 

Diagnosing hemobilia remains challenging due to its variable clinical presentations, ranging from asymptomatic cases to life-threatening hemorrhages. With mortality rates reaching 25% in untreated cases [6], timely and accurate diagnosis is critical. Despite advancements in imaging, no standard diagnostic protocol exists, complicating clinical decision-making. Although recent studies have highlighted diagnostic advancements such as CT angiography and MRI, no comprehensive review has integrated these findings with emerging therapeutic approaches like endoscopic hemostasis and embolization. This narrative review aims to provide a comprehensive overview of hemobilia, with a focus on diagnostic challenges, advances in imaging modalities, and evolving management strategies, including minimally invasive techniques.

## Review

Etiology and pathophysiology

Hemobilia, a rare but potentially life-threatening condition, arises from a diverse range of etiologies, including iatrogenic injuries, trauma [10], malignancies, infections, and vascular abnormalities. Each cause affects the biliary and vascular systems differently, leading to varied clinical presentations [7].

Iatrogenic Causes

Hemobilia contributes to approximately 65% of all major bleeding complications following hepatic trauma or interventional procedures, particularly those involving vascular injury within the biliary system [11]. Procedures commonly linked to this complication include percutaneous liver biopsy, diagnostic percutaneous transhepatic cholangiography (PTC), and percutaneous transhepatic biliary drainage (PTBD), most commonly due to vascular injury in the biliary system [12]. Other interventional procedures that may lead to hemobilia, though less frequently performed, include ultrasound-guided radiofrequency ablation, transarterial chemoembolization, and transjugular intrahepatic portosystemic shunt (TIPS) placement, which may result in the formation of fistulas between damaged blood vessels and the biliary duct [13]. While these procedures carry a higher risk, they represent a smaller proportion of cases due to their relative infrequency. However, the incidence of hemobilia related to procedures such as PTBD and transarterial chemoembolization has increased with the rising use of minimally invasive techniques for both diagnostic and therapeutic purposes [5,12].

Neoplastic Causes

The most common spontaneous cause of hemobilia is primary or metastatic hepatobiliary malignancy, which accounts for approximately 10% of all hemobilia cases [14]. Malignancies predispose to hemobilia by eroding fragile vasculature within the liver or bile ducts, causing spontaneous hemorrhage. Tumor invasion can disrupt normal vascular integrity, increasing the risk of both arterial and venous bleeding [15]. Although endovascular procedures are commonly used to treat hemobilia, they can sometimes result in hemobilia as an unintended consequence [2].

*Infections and Other* *Causes*

**Figure 1:**
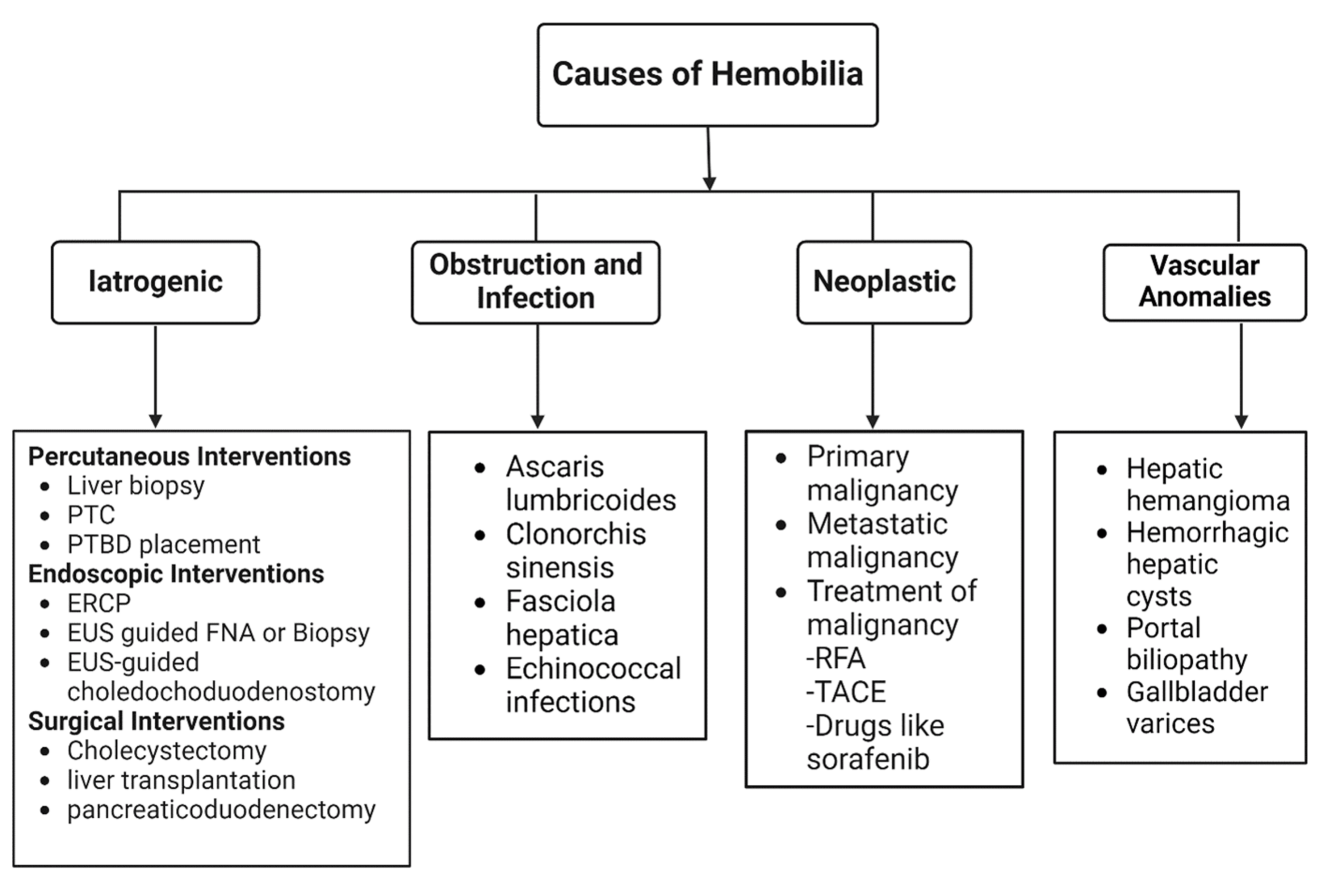
Causes of hemobilia. PTC: percutaneous transhepatic cholangiography; PTBD: percutaneous transhepatic biliary drainage; ERCP: endoscopic retrograde cholangiopancreatography; EUS: endoscopic ultrasound; FNA: fine needle aspiration; RFA: radiofrequency ablation; TACE: transarterial chemoembolization. [5,12,14,16,17,20] Figure created with BioRender. All credits to Aarfa Devani, September 2024.

The most common infectious causes of hemobilia include *Ascaris lumbricoides* (roundworms), *Clonorchis sinensis* (Chinese liver fluke), and *Fasciola hepatica* (sheep liver fluke). These parasites can obstruct the bile ducts, causing inflammation and subsequent erosion into nearby vasculature, leading to hemorrhage [16,17]. Less common causes of hemobilia are associated with vascular anomalies. These include hepatic hemangioma, intracholecystic Dieulafoy lesions, and hemorrhagic hepatic cysts [18]. Additional rare vascular causes include portal biliopathy and gallbladder varices [19,20]. Figure 1 provides a summary of the most common causes of hemobilia.

Pathophysiological mechanisms

Hemobilia is characterized by abnormal communication between the biliary and vascular systems, often resulting from trauma during invasive procedures. This can lead to immediate bleeding from fistulation between these systems [1]. In some cases, delayed bleeding may occur due to the formation of a pseudoaneurysm in a nearby vessel, which can eventually rupture, extending the interval between trauma and the manifestation of hemobilia symptoms [21,22].

Arterial hemobilia is more common and arises due to the high-pressure differential between the hepatic artery and the bile ducts [23]. Arterial-biliary fistulas, usually resulting from trauma or surgical procedures, lead to rapid bleeding and clot formation within the bile ducts. This can cause obstruction, ischemia, and inflammation [14,22]. Clinically, arterial hemobilia often presents as hematemesis or hematochezia [24]. Additionally, a sudden bloody output from biliary drains may indicate hemobilia. Delayed bleeding in arterial hemobilia can be caused by pseudoaneurysms or chronic erosion of the bile duct [21,23,25].

Venous hemobilia is less prevalent and typically originates from the portal or hepatic veins. This form of hemobilia is characterized by slower, less forceful bleeding due to the lower pressure in the venous system [14]. Unlike arterial bleeding, venous bleeding is more likely to stop spontaneously [23]. In cases of portal hypertension, increased pressure within the portal venous system can lead to the formation of varices that may erode into the bile ducts, causing hemobilia [26]. The gradual nature of venous bleeding often leads to delayed diagnosis, increasing the risk of complications such as secondary biliary cirrhosis or chronic inflammation [27].

Coagulation and Inflammation

Blood clots in the biliary tree can cause mechanical biliary obstruction, symptomatic jaundice, and right upper quadrant or epigastric pain. Thrombin produced during clot formation activates protease-activated receptors (PARs) [26], on endothelial cells and platelets, triggering the release of pro-inflammatory cytokines like IL-6 and TNF-α [23], This cytokine cascade not only exacerbates endothelial damage but also perpetuates clot formation, leading to recurrent biliary obstruction and progressive tissue damage within the biliary tree. Specific signaling pathways, such as JAK/STAT for IL-6, further amplify the inflammatory response, leading to continued tissue damage and clot propagation [14,24]. Ongoing bleeding within the biliary tree can lead to chronic biliary obstruction and potentially irreversible damage to the biliary epithelium [26].

Diagnosis

Hemobilia is a rare but serious condition, and the classic presentation is formally known as Quincke’s triad, which is a combination of jaundice, right upper quadrant abdominal pain, and upper gastrointestinal hemorrhage. However, the presentation of all three together only occurs in 22%-35% of the cases. Therefore, the absence of one of the symptoms makes early diagnosis quite challenging leading to delay in management [11,21,23,27].

The initial symptom of hemobilia often presents as right upper quadrant pain, which is typically intermittent and caused by biliary tract distention or obstruction by a clot [28]. If the condition progresses, obstructive jaundice can occur, leading to hyperbilirubinemia and potentially causing cholecystitis and cholangitis [29,30]. Hemobilia may cause abnormal liver enzyme elevations, particularly in serum bilirubin and alkaline phosphatase, with the degree of elevation depending on the severity of the obstruction caused by clots and blood. However, due to its rarity, other potential causes of elevated liver enzymes, such as dengue fever or herbal products, should be considered and ruled out through medical history and clinical evaluation [22,31-33].

Hemobilia may also present as hematemesis or melena due to bleeding from the biliary system into the duodenum. In more severe cases, massive hemorrhage can lead to hypovolemic shock [14]. Differentiating hemobilia from other causes of gastrointestinal bleeding, such as peptic ulcer disease, Mallory-Weiss tears, esophageal varices, and neoplastic lesions of the stomach or duodenum, can be difficult and requires ruling out these conditions [34,35]. Hemobilia should be considered in patients with unexplained gastrointestinal bleeding, particularly those with a history of liver trauma or biliary interventions [36-38]. When right upper quadrant pain and jaundice are associated with gastrointestinal bleeding, hemobilia becomes more likely, especially in patients with a history of liver trauma, biliary surgery, or invasive procedures such as liver biopsies [8]. Diagnostic imaging plays a key role in distinguishing hemobilia from other causes of gastrointestinal bleeding. The diagnostic process often begins with non-invasive techniques such as Doppler ultrasound, which can identify bile duct dilation and clots. For more detailed imaging, contrast-enhanced computed tomography or magnetic resonance cholangiopancreatography may be used. To visualize blood in the bile ducts and identify the source of the bleeding, especially if the source remains unclear, angiography provides clear vascular mapping. It is considered the gold standard for both diagnosis and therapeutic intervention in hemobilia. However, despite the benefits of ultrasound in life-threatening situations, its accuracy remains operator-dependent, requiring adequate training for physicians [39-41]. Endoscopic exploration may show bleeding in the duodenum, but it might not pinpoint the exact source. If a bleeding site or blood clot is identified during endoscopy or imaging, it can help distinguish hemobilia from other causes [42].

Diagnostic Modalities

Integrating imaging findings with clinical symptoms and other diagnostic tests provides a more accurate and comprehensive approach to diagnosis. This method ensures that treatment is tailored to each patient's specific needs, improving overall outcomes. Diagnostic approaches include various imaging techniques and endoscopic methods. 

Angiography is considered the gold standard for diagnosing hemobilia due to its unmatched ability to visualize vascular anatomy and pinpoint bleeding sources [5]. It is often used alongside other imaging techniques, such as computed tomography (CT) and endoscopic retrograde cholangiopancreatography (ERCP), to provide a comprehensive assessment of hemobilia. While CT offers initial diagnostic clues, and ERCP helps visualize and manage biliary obstructions, angiography provides detailed vascular information needed for definitive diagnosis and targeted treatment. Additionally, angiography is instrumental in the therapeutic management of hemobilia. Endovascular interventions, such as embolization, can be performed during angiography to control bleeding in hemobilia and other hemorrhagic conditions [43]. This procedure involves the selective occlusion of the bleeding vessel with embolic agents such as coils or particles, effectively stopping hemorrhage and reducing the need for invasive surgery [44]. 

Ultrasonography is invaluable as the first-line imaging modality for quickly assessing patients with suspected biliary colic. It offers real-time visualization of bile duct dilation, clots, and vascular abnormalities such as pseudoaneurysms [45]. However, its diagnostic accuracy may be limited by operator experience and patient factors such as obesity, making it less reliable in identifying the source of active bleeding [22,46]. Magnetic resonance cholangiopancreatography (MRCP) is particularly useful for detecting blood clots and assessing biliary obstructions with high-resolution imaging, making it an invaluable tool when ultrasound findings are inconclusive [47]. However, its high cost and limited ability to assess surrounding vascular structures often restrict its use in routine clinical practice [48]. Computed tomography (CT) scans are frequently the first imaging modality used due to their availability, speed, and effectiveness in visualizing acute hemorrhages. CT offers detailed cross-sectional images that can reveal blood within the biliary tract and identify associated complications, such as hepatic or biliary tract injuries [22]. Despite its utility, CT may yield non-specific findings, requiring additional imaging techniques or clinical correlation to confirm hemobilia. While CT is effective in identifying large volumes of blood and major vascular injuries, it may not always detect smaller or less obvious sources of bleeding [48].

ERCP serves both diagnostic and therapeutic roles, enabling the identification of bleeding sites and the management of obstructions. It can facilitate interventions such as stone removal or stent placement. ERCP findings in hemobilia may include amorphous, tubular, or cast-like filling defects, often associated with unexplained common bile duct or peri-hilar ductal dilation [22]. Endoscopic ultrasound (EUS) is gaining recognition for its role in assessing biliary tract lesions. It is particularly useful when other imaging methods, including ERCP and CT, fail to detect the source of obscure hemobilia [42]. The diagnostic modalities are summarized in Table 1.

**Table 1:** Comparison of diagnostic modalities for hemobilia. USG: ultrasonogram, MRI: magnetic resonance imaging, MRCP: magnetic resonance cholangiopancreatography, CT: computerized tomography, ERCP: endoscopic retrograde cholangiopancreatography. [5,22,42,44,45,47,48]

Modalities	Sensitivity	Specificity	Advantages	Disadvantages
USG	Mid-to-High	Low	Noninvasive. Useful for cases with low suspicion. Cost-effective.	Limited in obese patients. Findings are non-specific. Limited biliary duct visualization.
MRI/MRCP	Varies	Mid-to-High	Noninvasive. Visualization of biliary tree.	Requires more time for image processing. Costly. Prone to motion artifact.
CT	Mid-to-High	Low	Noninvasive. Fast and widely available.	Non-specific findings.
CT angiography	High	High	Gold standard, useful in management as it allows for planning endovascular interventions.	Invasive. Not recommended as first-choice for cases with low suspicion.
Endoscopy/ERCP	Low	High	Visualization of biliary tree.	Invasive. High capital expenditures.

Management

Management of hemobilia should begin with a systematic approach, such as the ABCD Primary Trauma Survey. Hemodynamically stable patients with nonpersistent mild bleeding can be treated with supportive care, avoiding surgical intervention [21]. Oral anticoagulants use should be evaluated and reversed, and crystalloid use should be limited, as indiscriminate use is associated with dilutional coagulopathy [49]. Blood transfusions should follow a 1:1 ratio of fresh-frozen plasma to packed red blood cells [50]. Conservative treatment focuses on resuscitation and correcting conditions like acidosis, hypothermia and coagulopathy, particularly in trauma patients, while being mindful of potential blood transfusion complications [20,51,52]. In some cases, mild bleeding is attributed to iatrogenic vein injury. In such instances, exchanging the PTBD with a larger catheter and adjusting its position can correct hemobilia through a tamponade effect [53]. Most intrabiliary blood in these patients will dissolve with bile flow [21]. However, in some cases, blood may remain in the bile ducts and coagulate, causing biliary obstruction. Reports of cholecystitis caused by such blockages, known as hemocholecystitis, are rare but carry an increased risk of mortality [21,54-56]. For secondary biliary obstruction, common procedures include nasobiliary drainage or ERCP with sphincterotomy and ductal clearance. Additionally, there are successful reports of clot dissolution with the use of intrabiliary alteplase [21,57].

Initial evaluation should focus on resuscitation and stabilizing the hemodynamic status [58]. If conservative management is selected, it should follow protocols similar to those for general gastrointestinal bleeding. This includes peripheral IV establishment, individualized fluid resuscitation, blood transfusion, and laboratory testing, including hepatic profile with bilirubin levels, alkaline phosphatase, gamma-glutamyl transferase, group testing and especially coagulation test. If an altered coagulation test is found, primary cause should be sought and reversed promptly, although coagulopathies are rare causes of hemobilia [14,57].

Endoscopic and Radiologic Interventions

Management of hemobilia consists of two main objectives: achieving hemostasis and maintaining bile flow [12,14,59]. While many endoscopic techniques have shown efficacy in treating general gastrointestinal bleeding, there is limited evidence specifically supporting their use in hemobilia. Further research is needed to establish the best practices for these techniques in the context of biliary bleeding [60,61]. Endoscopic techniques include sphincterotomy, stent placement, and direct hemostasis using methods such as clips or thermal coagulation. Sphincterotomy helps move blood clots from the bile duct to the duodenum, reducing pressure in the biliary tree and resolving obstructions. Stent placement may prevent recurrent obstructions by keeping the ducts open [62]. Endoscopic interventions are crucial in managing hemobilia, particularly when bleeding occurs within the bile duct. Techniques like endoscopic clipping and thermal coagulation (e.g., argon plasma coagulation) [63], are used to control bleeding vessels directly within the biliary ducts or at the ampullary site. These methods are effective in stopping hemorrhage in general gastrointestinal bleeding syndromes, but their specific efficacy in hemobilia has not been well-established [64].

For hemodynamically stable patients with hemobilia, especially when noninvasive imaging fails to identify a clear source of bleeding, upper endoscopy and ERCP are particularly valuable. ERCP not only allows for visualization of the biliary tree but also facilitates therapeutic interventions such as sphincterotomy, stent placement, and clot extraction [65]. These procedures help clear residual blood or clots from the bile ducts, reducing the risk of complications like cholangitis or biliary obstruction [66]. However, while these techniques are widely used, further research is needed to confirm their effectiveness specifically in the context of hemobilia [67,68].

Angiography with transcatheter arterial embolization (TAE) should be considered as the initial therapy of choice for treating hemobilia. TAE involves the selective catheterization of the bleeding vessel, followed by the administration of embolic materials to occlude the vessel and therefore stop the bleeding. The success rate of TAE is as high as 80%, making it one of the methods considered best in controlling bleeding [69,70]. TAE must be avoided in patients with liver allografts, cirrhosis and concurrent shock or portal vein thrombosis because they have poor collateral blood flow via the portal vein, making them susceptible to ischemic liver injury due to TAE [8,60,71]. These patients should use arterial stenting as a tamponading measure instead. TAE requires careful choice of embolic materials for the technique to be successful. Larger vessels are using coils, while distal embolization in smaller vessels is using polyvinyl alcohol (PVA). For quick and efficient embolization during bleeding within a tissue, n-butyl cyanoacrylate (NBCA) can be used [72,73].

Surgical Management

Although surgical management was the most predominant procedure done in these patients, endoscopic and radiological interventions have relegated it as a last-resort option. Surgical intervention carries a high mortality rate up to 10% [25,53]. Therefore, surgical management is indicated when there is a failure of conservative, endoscopic and radiologic interventions [10,74]. Other indications are major hemobilia, extensive liver trauma, hemodynamic instability, hemocholecystitis, infected or compressing pseudoaneurysms, altered anatomy such as in liver transplantation or tumor invasion [75], and bleeding refractory to alternative interventions [10,21,53].

Surgical management focuses on correcting the abnormal fistula between the splanchnic vessel and the intra/extrahepatic biliary system [74]. Various surgical modalities are used such as pseudoaneurysm repair, identification with selective ligation, and nonselective arterial ligation when a bleeding vessel cannot be identified (most frequently right hepatic artery) [21,76], to achieve hemostasis. Segmental liver resection is left as last resource option in rare cases where alternative modalities are insufficient [21,76]. Commonly cholecystectomy is not recommended unless there is existence of cholecystitis, or the gallbladder is involved [53]. After hemostasis is achieved the attention focus changes to clear the remaining blood inside the bile ducts. To achieve this, endoscopic interventions are generally necessary, patients that are not candidates for endoscopic procedures should be offered PTBD [20,58].

Future directions

Advances in diagnostic techniques are crucial for improving early detection and management of hemobilia. Modern imaging methods, such as CT with angiography, multispiral CT, and ultrasound with duplex scanning, are effective in identifying rare vascular pathologies causing hemobilia [56]. Dual-energy CT and enhanced MRI protocols are under investigation to enhance early detection and minimize diagnostic errors [77]. Emerging technologies, like virtual non-contrast reconstructions in CT, offer improved visualization while reducing radiation exposure [5]. Additionally, multiphasic MRI, despite being more time-intensive, provides a superior assessment of biliary obstruction and may benefit from future cost and time reductions [5]. The role of endoscopic ultrasound (EUS)-guided therapy is also expanding, particularly in patients with ambiguous etiologies or those unsuitable for invasive procedures [5]. Clarifying procedure-related risk factors and improving meta-analyses can help reduce morbidity and mortality rates following biliary interventions [78,79].

Future management strategies for hemobilia are evolving towards more targeted and minimally invasive approaches. Catheter angiography is currently a cornerstone for both diagnosis and treatment. This modality may benefit from novel embolic agents and vascular stents that enhance safety and efficacy while preserving hepatic arterial flow [5,77]. Endovascular embolization using coils and substances is an effective method, particularly when guided by a multidisciplinary approach [56]. New endoscopic techniques, including the use of dilute epinephrine, fibrin sealants, thermal coagulation, clipping, balloon tamponade, and stent placement, show promise in reducing the need for surgical intervention [5]. The development of EUS-guided interventions and alternative endoscopic therapies represents another key area of innovation [77]. Further research is needed to identify procedure-related risk factors, optimize management strategies, and standardize protocols across different clinical settings according to the needs of this new decade [78,80].

Significant gaps remain in the understanding of hemobilia, including the need for biomarkers to rapidly differentiate it from other forms of upper gastrointestinal hemorrhage [5]. Research is also needed to standardize treatment protocols, evaluate long-term outcomes of new embolic materials, and compare various imaging and interventional strategies in diverse clinical contexts [77,78]. Focus should be on long-term outcomes of minimally invasive techniques and developing predictive models for high-risk patients.

## Conclusions

Hemobilia is a rare but potentially life-threatening condition arising from various causes such as trauma, malignancy, and iatrogenic factors. Its clinical presentation is often subtle and varied, complicating early diagnosis, especially since Quincke's triad is not always present. A high index of suspicion is crucial for accurate diagnosis, particularly in patients with a history of liver trauma or biliary interventions. Advances in diagnostic imaging, including Doppler ultrasound and contrast-enhanced CT, are essential for identifying the condition and understanding its pathophysiology. Effective management of hemobilia requires a dual focus on achieving hemostasis and maintaining bile flow. Endoscopic techniques, including sphincterotomy, stent placement, and direct hemostasis methods like clips or thermal coagulation, are pivotal in addressing the cause and severity of the bleeding. ERCP is particularly effective in identifying the bleeding source, treating it, and clearing blood clots from the bile ducts, thereby preventing complications such as cholangitis and biliary obstruction. Early recognition and timely intervention are key to preventing severe complications and improving patient outcomes in hemobilia.
